# An Evolutionary Point of View of Animal Ethics

**DOI:** 10.3389/fpsyg.2020.00403

**Published:** 2020-04-02

**Authors:** François Criscuolo, Cédric Sueur

**Affiliations:** ^1^Université de Strasbourg, CNRS, IPHC UMR 7178, Strasbourg, France; ^2^Institut Universitaire de France, Paris, France; ^3^CEERE, Centre Européen d'Enseignement et de Recherche en Ethique, Université de Strasbourg, Strasbourg, France

**Keywords:** animal ethics, evolutionary biology, trade-offs, human-animal relationships, environmental ethics, empathy, sentience, one health

## Introduction

The observation that animals may respond to the emotional states of conspecific or even heterospecific individuals is not new. Darwin broached the question by underlying the ability of animals to express sympathy, i.e., the response to non-self-emotional status, even across species barriers. More importantly, he tried to find the evolutionary origin of this animal trait, suggesting that it evolved from the selective advantages of kinship behavior in the struggle for life (Darwin, 1872). Such a behavior corresponds, for instance, to alloparental care, which is relatively common in mammals and birds and is now also characterized in fishes and insects (Josi et al., 2019; Wu et al., 2020). After more than one century, the need to define what exactly non-human animals are able to feel and—from this starting point—rethink the legal status and place of animals in human societies is becoming increasingly necessary. This can mainly be considered as an indirect consequence of people's increasing awareness of the consequences of dramatic human-driven impacts on the global climate and biodiversity, but this also holds true for the daily issues concerning animal life and welfare. However, because assessable currencies are required to establish laws, animals were classified into categories based on ecological (e.g., invasive species, pest, wild, and domestic), biological (e.g., vertebrates and invertebrates), or cognitive (e.g., primates and cephalopods) traits. This should help lawyers to define ethical rules of animal use by humans and, from that, determine the rights of animals (Rollin, 2006; Donaldson and Kymlicka, 2011). A major issue of such an approach to animal ethics is, however, that it remains human-centered (i.e., anthropocentrism) and focused on human thought (i.e., anthropomorphism). Indeed, the human empathy tree appears to be different to the phylogenetic tree, meaning that human empathy toward other organisms is not equally distributed within the tree of life (Miralles et al., 2019). Why, for instance, are cognitive capacities considered to be highly important in defining which animals can be used for human benefit? Why are individual lifespans or animal culling considered to be the most important parameters in the ethical equation? This is all because these criteria are what define us, citizens of modern human societies, as the superiors. We project our wishes and expectations regarding longevity, issues of euthanasia, and the death penalty onto animals.

Due to the varied cultural differences in human society, there exists a large panel of moral intuitions, and social activism for animal rights has increased for multiple reasons (Herzog and Golden, 2009); however, most of these keep in mind common currencies of human morality. While, in Biology, an animal is defined as a heterotrophic multicellular organism, its legislative definition is more restricted to vertebrates or domestic animals. However, if we want to legislate animal rights in an unbiased way, a non-anthropocentric definition of animal beings—and, more generally, all living beings—should be established, and in biology nothing has sense out of evolution (Dobzhansky, 1973). In this article, we aimed to raise the incongruity of defining animals from a human point of view. Indeed this point of view, while being understandable due to anthropomorphism, does not take into account what evolution could to tell us about ethics. We have proposed an alternative non-anthropocentric view of how thinking of animal beings from an evolutionary perspective may help us to redefine animal ethics. Evolutionary ethics defines how we should behave with relation to human heterospecific living beings by freeing us from human-based cultural or emotional considerations. We have proposed that animal ethics (principally based on individuals) cannot be distinguished from environmental ethics, and evolutionary ethics may therefore also help us to solve the paradoxical position of humankind concerning biodiversity; we know that we are doing wrong, but, by doing wrong, we have promoted, thus far, the survival and propagation of our own species. Exactly what evolution actually focuses about? Evolutionary ethics is thus also concerned with human evolution and promotes the exit of humanity from the evolution paradigm.

## Animal Ethics From a Human Perspective

### Ethical Considerations

Animal ethics (animalism) usually differs from environmental ethics (environmentalism). The first one is supposed to be concerned with defending animals as individuals and caring about their use and welfare. The second one defends animals as species and their related environments. For instance, the environmentalist school of thought would consider the eradication of cats as an invasive species killing endemic ones in Australia, but the animalist school of thought would not. The two ethics are, however, becoming increasingly connected due to the complex consequences of human activities. For instance, recent Australian mega-fires have raised both environmental and animal ethical issues (Nolan et al., 2020). Indeed, it is likely that there are two main reasons that have led to the increased demand from citizens, at least in more economically developed countries, to change the policy defining our relationship with the animal world. The first one is related to the fact that animals and their ecosystems actually form a whole functional entity, human species included. The accelerated sixth mass extinction of the Earth's history (Ceballos et al., 2017), related to the domination of humans over most ecosystems, directly threatens human civilization *via* its impact on ecosystems' viability. For scientists, the fact that biodiversity is the cornerstone of the stability and productivity of ecosystems has been recognized for a long time, and has become an important discipline within the field of ecological research (Tilman, 2000). This is not a scientific claim based on theoretical considerations or modeling, but it results from the accumulation of experimental and observational evidence (Bolund and Hunhammar, 1999; Worm et al., 2006; Zhang et al., 2007). Decision making regarding protection laws have necessitated a demonstration of the decreased biodiversity that has endangered numerous ecosystem services, including food production, fresh water filtering, or waste recycling, all of which are of tremendous importance for human survival. However, because human activities are harmful toward biodiversity but are also largely beneficial in the short term for human society (e.g., politics supporting rapid economic growth), there has had to be a trade-off between economics and ecology (e.g., Varijakshapanicker et al., 2019). Thereby, the rights of wild animals, but also those of farmed animals, have been taken into account so far in the human-biased point of view, and this has largely been based on the valuable societal benefits they provide. However, animals belong to ecosystems, and they cannot therefore be considered separately from issues of ethics. They consequently stand at the collision point between our own survival considerations (the most profitable and productive ways to exploit animals) and purely ethical considerations (the limits of our exploitation of animal resources), leading to a split between animalism, abolitionism, and welfarism.

### Fundamentals of Morality

The second reason for changing our behavior toward animals is based on the special position humans have granted themselves in the tree of life (which is actually an assemblage of bushes of life and a non-directional evolutionary process) (Rokas and Carroll, 2006) as being the only species with enough cognitive capacities to think for others. The rules established so far to decide what can and cannot be done with animals have inevitably followed the subjective feelings of humans, though only, of course, to a certain extent. Bentham's question of “can they suffer[?]” is not human-related but “suffering-related” (Nussbaum, 2004), even if humans also experience pain and suffering. If it is indeed “a non-sense situation if we want to establish [all] rules of animal ethics based on human morality concepts,” the question of “can they suffer[?]” is *not* a human morality rule but a broad biological one. This should be recognized, but the fact that it is human-centered to give less importance to arthropods, such as manta, spiders, ants, or bees, should also be recognized; they sacrifice their own lives and are eaten alive for the sake of the group and species. Such (mainly moral) concerns, for instance regarding cruelty toward animals, were first presented in ancestral religious texts, but the underlying reasons remain that cruel behaviors toward animals may be extended if not punished toward human conspecifics (Rollin, 2006). This human-centered questioning regarding animals—including the issue of the definition of an animal, e.g., vertebrates vs. insects and others (see House, 2018)—has prevailed, even with the recognition of animal rights in society. For instance, the codification of animal use in the mid-twentieth century was first restricted to non-human primates, excluding mice and rats (despite representing >90% of the animal models for scientific research) and farmed animals (for obvious economic reasons, which could also be interpreted as evolution-derived decisions to maximize human fitness). More recently, legislation was extended to vertebrates in general (birds, anurans, fishes, etc.) and cephalopods (Hartung, 2010). Still, what are the bases for this discrimination between animals with rights and others? Because being requires thinking as well as just feeling, animals that retain some cognitive abilities can be classified as “human-like” and can then benefit from rules protecting them. Such animal awareness or consciousness (i.e., sentience, the ability of animals to be conscious of suffering) was established by cognitive ethologists from comparative studies of behavioral and neuroanatomy homologies between animals and humans (Allen and Bekoff, 2007). An additional drawback to applying human morality to animals is that animals are amoral beings. For instance, predation, infanticide, and forced copulation have all coevolved with numerous animal life-history strategies because they may promote individual fitness, which is the driving force of species evolution. We are facing a non-sense situation if we want to establish rules of animal ethics based on human morality concepts. One may ask “what is the meaning of morality from a human frame of reference”? Humans are certainly in an unbalanced system conducting to issues for all living beings, humans included. Even without morality, species live in stable ecosystems through the use of evolutionary stable strategies [ESS, (Taylor and Jonker, 1978)]. This principle of ESS is now applied to human activities without referring to morality but with more focus on balanced systems (He et al., 2019).

### Extending Ethics to Non-animal Beings

One clear dichotomy in humans is the distinction we make between animals and non-animals, such as plants or mushrooms. This distinction is based on clear morphological, physiological, and ecological traits as well as evolutionary origin but also, for the purposes of ethics, on the capacity for suffering or of sentience. To our knowledge, no study showed that plants are sentient. Plants or mushrooms have no organs with which to centralize information and create or process mental states (e.g., feelings) (Calvo et al., 2017; but see Pelizzon and Gagliano, 2015). However, this conceptualization of sentience as something needing centralization of information is quite human-centered. Even if plants and mushrooms are not capable of sentience, they are at least capable of reception and integration of different information sources [e.g., chemical, visual, and tactile (Trewavas, 2016; Calvo et al., 2017)]. Some recent studies have shown that they are able to learn, react to mechanical stress, and even communicate (Poelman et al., 2012) about this stress (Khait et al., 2019). Of course, we do not say that a plant is akin to a vertebrate or even an insect, but the ethical dichotomy we make between animals and plants appears so far to be too simplistic. Again, the evolution of the perception of humans (anthropocentrism and anthropomorphism) may have favored this ethical dichotomy.

### Human Interests in Ethics

This is certainly a very rough picture of the present debate on animal ethics, but it appears to be in line with an issue strictly related to humans and to human fitness (i.e., the growth rate of the human population). In fact, by following this way of thinking, we are trapped in an equation that has been simply resolved so far by natural selection, which sees human fitness as paramount. This has worked very well since the human population has never been so large, and the quality of living conditions has also improved exponentially over the last century. Humans care about animal ethics once their own ethical issues are resolved. The evolution of civilizations shows different steps in human morality; first came the abolition of slavery, and this was followed by gender equality, children rights, and then animal ethics. This means that only once human populations have reached an upward threshold level of life quality may they care about the well-being of other species. This process might be thought of in terms of fitness too, and we may wonder whether reviving human interests in animal ethics is not fitness-oriented due to the challenges imposed by the global changes. For instance, when the use of biodiversity endangers human health, animals may be better protected, such as through the wildlife trade and animal protection policies in China, which will likely be more regulated in light of the recent SARS or Covid-19 spread (Bell et al., 2004; Bonilla-Aldana et al., 2020; Hemida and Abduallah, 2020). The only limit to human fitness is imposed by the environment, and the forthcoming consequences of global warming will largely be deleterious for human populations (Burke et al., 2017). Because of that, we need to get out of the evolutionary trap of animal ethics as it is currently imposed by its anthropocentric definition.

## Animal Ethics From an Evolutionary Perspective

### Interconnected Species

We are living in a world that hosts an incredible diversity of life forms, from invisible unicellular organisms to plants and to huge marine vertebrates. Earth biodiversity even transports us to old ages *via* the continuous discovery of incredible fossils of all forms. Life on Earth, then, first refers to the past, and the functioning of the current ecosystem is the result of a rich history of co-evolution that is 3 billion years old. This is the very first and most important fact to recognize when trying to escape the human perspective of animal ethics. One first consequence is that the ethics of animals is not different from the ethics of ecosystems because all species have evolved as interconnected entities (Thébault and Fontaine, 2010; Ulanowicz et al., 2014), and if one is granted rights, the second automatically obtains the same rights. This might happen directly, with animals and plants being parts of the ecosystems, or indirectly, such as protecting a flag or umbrella species, e.g., the giant panda, for the protection of the all ecosystems (Shen et al., 2020). Granting legal identity to rivers protects more than just the rivers themselves; it also protects biodiversity, cultures, and ethnicities (Wilson and Lee, 2019). Respect is universal; it is not limited to our needs or feelings. Distinguishing between animals and their environments is merely driving down the road of domestication and will transform animals into non-evolutionary objects.

### Domestication and Ethics

From our point of view, pets are the most common representation of non-evolutionary animal objects, being entirely integrated into the human ecosystem and our morality rules, and it is not surprising that they are the first animals to be granted animal rights. Domestication is not totally aberrant in the context of evolution because it has been beneficial both to humans (i.e., mainly in the production of food) and to animal species, which has succeeded in terms of diversification (to a point, see Destoumieux-Garzón et al., 2020), survival, reproduction, and population dynamics. One can see farming as a human–animal symbiosis: it is good for all at the species level. The application of evolutionary questioning to production science actually opens up for interesting avenues of applied research to improve the living conditions of farming animals and better define their ability to adapt to the current environmental changes (Destoumieux-Garzón et al., 2020). The latter is exactly what the current laws of animal ethics want to rule out. Reducing animals to objects (“good” like pets or “bad” like potential human predators or competitors) has at least two drawbacks: it favors anthropomorphism and annihilates the reality of non-human living beings, thereby justifying human overexploitation of ecosystems. In addition, it intuitively places humans as the drivers of the future evolution of animal species and strengthens the idea that humans do not actually belong to the animal kingdom, and all this for insufficient reasons, such as specific evolutionary history (i.e., granting us with an exceptional cognitive capacity).

### Replacing Human Activities With Ecosystems

In fact, trying to define and categorize animals using the consciousness or the animal sentience argument (even through the precautionary principle, Birch, 2017) could largely be attributed to the self-proclamation of humans as superior organisms. By accepting this, however, we forget that evolution is a random process with no directionality or final objective (of which the final objective is certainly not the creation of *Homo sapiens*). Moreover, humans often try to distance themselves from what they call *nature—*creating a binary between nature and culture, urban environments and natural ones. They do not feel belonging anymore to where they come from. Nature is a concept, an abstraction invented by humans that allows us to establish a distance between ourselves and non-humans so as to better dominate them (Descola, 2019). Replacing the human–animals/ecosystems relationship in the context of symbiosis (i.e., equality of species in relation to benefits) and applying it to wild species will naturally help us to redefine but also accept the rights of animals and all livings beings (the main right being to live freely) merely by recognizing their role in the global functioning of the environment we are living in. For instance, humans have always accelerated the extinction of large animals because they represent a threat to humans and our livestock (Haynes, 2018), thereby favoring human and livestock population growth. The current issue represented by the population dynamics of large predators in modern countries is mainly discussed within the context of the economics of livestock management. However, we could also consider livestock as potential prey interacting with predators and try to select for appropriate anti-predator behaviors (Frid, 1997; Moreira-Arce et al., 2018) that may reduce the economic costs (and, perhaps more importantly, the bitterness of farmers) within acceptable limits. These limits should not apply only to parts of the society that are the more exposed to animal interactions (farmers), and ethical efforts should concern public research, the food-processing industry, and citizens in general.

### Symbiosis Over Exploitation

Nevertheless, humans may consider themselves as a superior species for good reason; it may help us to reassess human fitness through the regulation of human population dynamics. This remains the only way to reallocate environmental space to animals and to reduce global warming, i.e., to define evolutionary animal ethics. Adopting rules that will lead to a decrease in the human population is a painful renunciation of our selected inclination toward increased individual fitness. While being a crucial step for the planet, this Malthusian theory (Chu and Tai, 2001) remains the most difficult concept to explain to the population because it contradicts the optimal (and so far very successful) fitness trade-off of the human species that has been selected over thousands of generations. As such, it is written in our genes and holds a central place in our animal subconsciousness. Moreover, it also politically challenges the individual liberty of the life-history decisions of citizens. Still, such an evolutionary puzzle, like the reproduction/longevity trade-off (i.e., that which prevents simultaneous maximization of both traits), has previously been resolved by our species, as human are the only long-lived primate with high fertility (Walker et al., 2007). Moving away from a successful life-history strategy is a natural non-sense as well as a radical paradigm change for the entirety of society, and for these reasons it is likely to be a long-term objective that is incompatible with the environmental urgency of the twenty-first century. Nevertheless, it is up to our public authorities to launch the beginnings of such a political message and to find short-term alternatives, such as helping countries to define wildlife animal ethics, and linking it with immediate economic benefits could be one possible solution. It will be necessary to create a national index of animal biodiversity that corresponds to an internationally recognized economic value, each species being granted with a specific value based on its rarity, role in the ecosystem (including criteria for the attractiveness of ecosystems for the sake of tourism), as well as importance for scientific research and education. This would help to drive international policies for environmental protection and animal rights in relation to their economic payoffs.

## Conclusion

Animal ethics is a fundamental question for human beings, not because it promptly refers to animals but because it returns humans to their original roots. Because all life on Earth is the product of natural selection, humans are first defined by evolutionary trade-offs related to fitness. To maximize our survival, the environment has been anthropized, including animal species selection and control of population dynamics. To do this, we have also defined what animals should be. Rather than doing this, however, and by using evolutionary theory, we have suggested that we should make the ultimate human step to remove ourselves from the process of natural selection and escape the human focus on evolutionary trade-off optimization when helping to define what animals really are. This new evolutionary ethics thus proposes to halt the differentiation between animal ethics and environmental ethics and to replace human activities at the core of ecosystems. It is also a true ethical issue that belongs to economically developed countries in which human welfare has reached a sufficient level so as to make room for caring about animals and the environment on a global scale. These are the bases of evolutionary animal ethics.

## Author's Note

The idea for this opinion paper occurred during an animal ethics training session for scientists given by CS at the University of Strasbourg.

## Author Contributions

All authors listed have made a substantial, direct and intellectual contribution to the work, and approved it for publication.

### Conflict of Interest

The authors declare that the research was conducted in the absence of any commercial or financial relationships that could be construed as a potential conflict of interest.
